# A new analysis approach of epidermal growth factor receptor pathway activation patterns provides insights into cetuximab resistance mechanisms in head and neck cancer

**DOI:** 10.1186/1741-7015-10-43

**Published:** 2012-05-01

**Authors:** Silvia von der Heyde, Tim Beissbarth

**Affiliations:** 1 Department of Medical Statistics, University Medical Center Göttingen, Humboldtallee 32, Göttingen, 37073, Germany

**Keywords:** HNSCC, EGFR, cetuximab, drug resistance, matrix factorization, GSEA, pathway signature

## Abstract

The pathways downstream of the epidermal growth factor receptor (EGFR) have often been implicated to play crucial roles in the development and progression of various cancer types. Different authors have proposed models in cell lines in which they study the modes of pathway activities after perturbation experiments. It is prudent to believe that a better understanding of these pathway activation patterns might lead to novel treatment concepts for cancer patients or at least allow a better stratification of patient collectives into different risk groups or into groups that might respond to different treatments. Traditionally, such analyses focused on the individual players of the pathways. More recently in the field of systems biology, a plethora of approaches that take a more holistic view on the signaling pathways and their downstream transcriptional targets has been developed. Fertig et al. have recently developed a new method to identify patterns and biological process activity from transcriptomics data, and they demonstrate the utility of this methodology to analyze gene expression activity downstream of the EGFR in head and neck squamous cell carcinoma to study cetuximab resistance. Please see related article: http://www.biomedcentral.com/1471-2164/13/160

## Background

**Figure 1:**
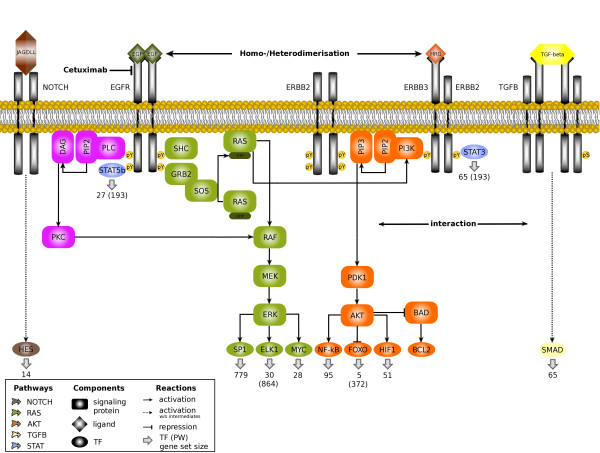
**Signaling pathways involved in head and neck cancer**. The main pathways contributing to signaling in head and neck cancer, that is, NOTCH, RAS, AKT, TGF-*β *and STAT, are depicted in an abstract manner including only most relevant cellular components in this context. Activation is induced via binding of ligands to extracellular receptor parts, resulting in intracellular phosphorylation cascades leading to transcription of certain gene sets (numbers correspond to gene set sizes in Fertig et al. [20]) related to individual transcription factors or whole pathways (total number of targets quoted in brackets).

The epidermal growth factor receptor (EGFR) is a transmembrane receptor belonging to the group of receptor tyrosine kinases that forward extracellular signals via phosphorylation cascades, which finally arouse cellular responses. This kind of proteins is often related to cancer due to mutations or overexpression leading to aberrant signaling and resultant excessive proliferation [1-3]. Main adaptors for EGFR are GRB2 and Shc, activating the mitogen-activated protein kinase (MAPK) pathway via RAS. ERBB2 binding sites are more promiscuous, enabling the respective dimers to activate not only the MAPK but also the phosphoinositide 3-kinase (PI3K) pathway, the two major pathways in ERBB signaling responsible for cell proliferation, cellular survival and anti-apoptosis [4]. Also, cross-talk of these pathways exists, offering potential bypass strategies in the protein network (Figure 1). Due to the association of overexpressed EGFR with poor prognosis of head and neck squamous cell carcinoma (HNSCC), cetuximab, a monoclonal antibody targeting the receptor, is applied in common therapeutic strategies [5]. However, many HNSCC patients are non-responders or develop resistance, which is suspected to result from aberrant activation of EGFR pathways [6,7]. To improve such a targeted therapy, it would be beneficial to gain insight into the individual molecular specificity of the targeted pathway per patient [8]. Thus, in a personalized medicine approach, the relevance of the pathway should be revealed in advance to treatment. Therefore, the detection of common gene activity patterns among sample subsets is used to stratify patients based on their gene expression profiles.

Gene expression microarrays are a widely used tool to measure genomewide transcription within cell lines or tissues under varying conditions. Usually, gene-wise statistical tests, for example employing linear models, are then performed to determine differentially expressed genes [9]. Methods to find overrepresentation of functional gene sets or pathway genes, so called gene set enrichment analysis (GSEA), are employed in order to interpret the resulting long lists of differential genes [10-12]. To monitor the activity of certain pathway parts or transcription factors (TFs), gene sets of TF target genes, as they can be retrieved from databases like TRANSFAC, are of special interest [13]. Another aspect of data analysis is revealing gene expression patterns of patient or gene groups by clustering or dimension reduction techniques [14]. A number of specialized methods have been proposed previously, for example, clustering genes and patients simultaneously into biclusters [15], applying predefined gene signatures in guided clustering approaches [16] or signal flow reconstruction in pathways from downstream effects of perturbation experiments [17].

Fertig et al. have proposed the new method Coordinated Gene Activity in Pattern Sets (CoGAPS) [18] and made it available as add-on for the popular free statistical computing software R [19]. It combines a matrix factorization technique with GSEA of downstream transcriptional targets to determine patterns of pathway activity. They now demonstrate its utility to study cetuximab resistance in HNSCC by analyzing gene expression patterns downstream of EGFR [20].

## Discussion

Fertig et al. present a modeling approach of cetuximab resistance mechanisms applying the CoGAPS algorithm to infer gene expression signatures, distinguishing five variants of HaCaT cell lines under different media conditions concerning serum starvation and addition of EGF or TNF-*α. *These immortalized keratinocytes are chosen as model systems as they are well characterized and their genetic aberrations reflect early oncogenic events in HNSCC. The detected pathway signatures are then used to compare two isogenic HNSCC cell lines, that is, UMSCC1 and 1CC8, of which the latter is known to be cetuximab resistant in contrast to the sensitive UMSCC1 cell line.

### The CoGAPS method

**Figure 2:**
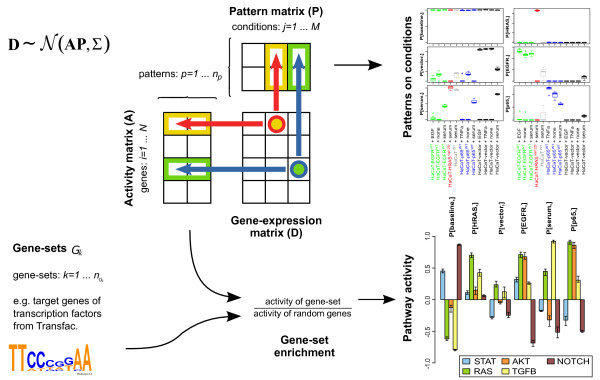
**Scheme of CoGAPS algorithm**. In the first step, CoGAPS factorizes the gene expression data matrix (D) into the amplitude (A) and pattern (P) matrices. The pattern matrix summarizes common expression patterns among different experimental conditions. The amplitude matrix summarizes the gene expression activity of all genes in the specified patterns. In the second step, the expression activity from matrix A is analyzed for pattern-specific pathway activation. This is done by testing for enriched activity of gene sets of transcription factor targets.

This method factorizes the input gene expression data matrix, with genes as rows and experimental conditions as columns, into two matrices, one defining different patterns of conditions and one storing amplitudes indicating the involvement strength of the respective gene in each pattern (Figure 2). The second step of CoGAPS then is to use the input list of gene sets, namely pathway-related TFs and their targets, to calculate *Z*-scores determining pattern-specific pathway activity. Thus, the required inputs are the mean gene expression data per experimental condition, the estimated standard deviations from replicates, a list of gene sets for TF targets, hyperparameters indicating the sparsity of the pattern and amplitude matrices and, finally, the number of patterns to be inferred. The outputs are the activity levels of the different experimental conditions per pattern, individual gene activity per pattern and enrichment scores for gene set activities in each pattern.

### Analysis of EGFR downstream activation patterns on HNSCC data

The HaCaT variants include transfected cell types overexpressing EGFR, NF-kappa-B p65 subunit or mutant HRAS. Transcriptional targets of sub-pathways under investigation belong to STAT, AKT, RAS, Notch and TGF-*β *due to their implication in HNSCC. Applying CoGAPS to the HaCaT gene expression data reveals six patterns, which separate the samples well according to their experimental conditions. Thus, the patterns are attributed to baseline HaCaT activity, HaCaT-HRAS^Val12^, HaCaT-vector control, HaCaT-EGFR^WT^, serum and HaCaT-p65^WT^. Afterwards, the activities of downstream transcriptional targets are calculated based on the *Z*-scores. This confirms upregulation of expected pathways but also indicates potential cross-talk mechanisms. The method is compared to a standard linear model approach with outcomes less consistent with prior knowledge. For example, CoGAPS reveals RAS and STAT overrepresentation for forced HRAS and EGFR expression in HaCaT cells and assigns Notch activity to the baseline pattern. Finally, the CoGAPS patterns are projected to the gene expression data of UMSCC1 and 1CC8 with and without cetuximab treatment. The most interesting finding here is that the pathway signature associated with HaCaT-HRAS^Val12 ^could predict the cetuximab treatment response, that is, treatment reduces the signature amplitude in sensitive UMSCC1, but not in resistant 1CC8. This is interpretable in such a way that cetuximab fails to repress the hyperactive RAS pathway in resistant HNSCC cell lines. A possible extension of this for the future would be to apply the learned signature to patient data and test whether it is likewise able to predict clinical parameters such as treatment response.

## Conclusions

The main drawback of established techniques to infer activity of gene sets, clustering for example, is that they are neglecting multiple regulation of genes, that is, gene re-usage and co-regulation by diverse pathways and TFs as well as coordinated activity of gene sets, for example, pathway cross-talk, which actually constitutes a specific phenotype. To overcome this disadvantage, the CoGAPS algorithm focuses on gene sets instead of isolated genes for inferring biological processes based on transcriptional data. The multitude of computational methods and tools analyzing activity patterns of (interacting) pathways should be further developed and compared to each other in the future. The presented results indicate the potential of the CoGAPS algorithm to detect transcriptional signatures as biomarkers for individual drug sensitivity or resistance, respectively. These signatures will have to be tested and prove their value in clinical practice in the future.

## List of abbreviations

EGFR: epidermal growth factor receptor; CoGAPS: Coordinated Gene Activity in Pattern Sets; HNSCC: head and neck squamous cell carcinoma; GSEA: gene set enrichment analysis; MAPK: mitogen-activated protein kinase; PI3K: phosphoinositide 3-kinase; TF: transcription factor.

## Competing interests

The authors declare that they have no competing interests.

## Authors' contributions

SH and TB contributed equally to this commentary. Both authors were involved in the development, writing and revisions of this manuscript. Both authors read and approved the final manuscript.

## Authors' information

SH is a research scientist focusing on network reconstruction from proteomics data and systems biology of the EGFR pathway in breast cancer. TB is an associate professor for statistical bioinformatics in the Department of Medical Statistics at the University Medical Center Göttingen. His main research focus is on the development of methods for the analysis and interpretation of high-throughput genomics data and on network reconstruction algorithms. He leads the multidisciplinary consortium BreastSys with systems biological analysis of the EGFR pathway as key aspect.

## Pre-publication history

The pre-publication history for this paper can be accessed here:

http://www.biomedcentral.com/1741-7015/10/43/prepub
